# Distinct effects of rectum delineation methods in 3D-confromal vs. IMRT treatment planning of prostate cancer

**DOI:** 10.1186/1748-717X-1-34

**Published:** 2006-09-06

**Authors:** Matthias Guckenberger, Jürgen Meyer, Kurt Baier, Dirk Vordermark, Michael Flentje

**Affiliations:** 1 Department of Radiation Oncology, University of Wuerzburg, Josef-Schneider-Str. 11, 97080 Wuerzburg, Germany

## Abstract

**Background:**

The dose distribution to the rectum, delineated as solid organ, rectal wall and rectal surface, in 3D conformal (3D-CRT) and intensity-modulated radiotherapy treatment (IMRT) planning for localized prostate cancer was evaluated.

**Materials and methods:**

In a retrospective planning study 3-field, 4-field and IMRT treatment plans were analyzed for ten patients with localized prostate cancer. The dose to the rectum was evaluated based on dose-volume histograms of ***1) ***the entire rectal volume (DVH) ***2) ***manually delineated rectal wall (DWH) ***3) ***rectal wall with 3 mm wall thickness (DWH_3_) ***4) ***and the rectal surface (DSH). The influence of the rectal filling and of the seminal vesicles' anatomy on these dose parameters was investigated. A literature review of the dose-volume relationship for late rectal toxicity was conducted.

**Results:**

In 3D-CRT (3-field and 4-field) the dose parameters differed most in the mid-dose region: the DWH showed significantly lower doses to the rectum (8.7% ± 4.2%) compared to the DWH_3 _and the DSH. In IMRT the differences between dose parameters were larger in comparison with 3D-CRT. Differences were statistically significant between DVH and all other dose parameters and between DWH and DSH. Mean doses were increased by 23.6% ± 8.7% in the DSH compared to the DVH in the mid-dose region. Furthermore, both the rectal filling and the anatomy of the seminal vesicles influenced the relationship between the dose parameters: a significant correlation of the difference between DVH and DWH and the rectal volume was seen in IMRT treatment.

**Discussion:**

The method of delineating the rectum significantly influenced the dose representation in the dose-volume histogram. This effect was pronounced in IMRT treatment planning compared to 3D-CRT. For integration of dose-volume parameters from the literature into clinical practice these results have to be considered.

## Background

Dose escalation has been effective in radiotherapy treatment of localized prostate cancer. Especially intermediate risk patients benefit from doses higher than 70Gy, whether low and high risk patients do so is controversial [1].

Late rectal toxicity, in particular late rectal bleeding, turned out to be the limiting factor in dose escalation [2]. The Patterns of Care Study stated that the incidence of severe rectal and bladder complications almost doubled when dose levels were increased beyond 70Gy with conventional treatment [3]. Three dimensional conformal radiotherapy (3D-CRT) in comparison to conventional radiotherapy resulted in lower rates of late rectal toxicity [4] and allowed the safe administration of doses up to 80Gy. Intensity-modulated radiotherapy (IMRT) has been indicated to be beneficial in comparison with 3D-CRT and made further dose escalation to 86.4Gy possible [5].

The improvements from conventional RT to 3D-CRT and from 3D-CRT to IMRT are due to more conformal dose distributions with the high dose region confined to the target volume and sparing of organs-at-risk [6,7]. The correlation between the volume of the rectum within the high dose region and the risk for late rectal toxicity suggested a dose volume effect [8].

Dose-volume histograms (DVH) are widely used to evaluate treatment plans and to estimate the risk for toxicity. For solid organs like most tumors, liver or parotid gland the DVH is based on the volume encompassed by the outer contour of the organ. For "hollow" organs like the rectum or bladder, the use of the DVH is controversial as this implicates that rectum and bladder are solid organs. From a radiobiological point of view the rectal wall without its filling defines the critical structure. The content of the hollow organ is irrelevant in terms of risk of complication. Therefore dose-wall histogram (DWH) and dose-surface histogram (DSH) have been suggested to describe the dose to hollow organs in a more appropriate way. Whereas DVH and DWH calculate dose distributions to 3D volumes (entire rectal volume and rectal wall respectively) DSH calculates dose distributions to 2D surfaces, e.g. the outer contour of the rectal wall.

This study compared and analyzed the dose distribution of the rectal DVH, DWH and DSH in 3D-CRT and IMRT treatment planning for prostate cancer. A literature review of the association of these dose parameters with late rectal toxicity was conducted.

## Materials and methods

This retrospective planning study included ten consecutive patients treated for localized prostate cancer at the Department of Radiation Oncology of the University of Wuerzburg, Germany, between August 2003 and November 2003.

A spiral planning computed tomography (CT) scan was acquired in the supine position. Slice thickness was 5 mm. Patients were advised to have an empty bowel and a full bladder. A full bladder was advised to keep larger parts of the bladder outside the treatment fields. Simultaneously, a distended rectum has been demonstrated to be not reproducible during the total time of treatment [9]. Patients with a distended rectum in the planning CT received a second CT study in the first or second week of treatment. If the rectal filling was significantly smaller, a new treatment plan based on the second planning CT was generated.

Oncentra™ Treatment Planning (OTP) Version 1.3 (Nucletron, Veenendaal, Netherlands), now Masterplan™, was utilized for treatment planning.

The clinical target volume (CTV) encompassed the prostate gland and seminal vesicles to simulate treatment plans with high risk of vesicle involvement. This target volume concept was used because IMRT is particularly beneficial for concave targets wrapped around organs-at-risk (OAR) [10]. The planning target volume 1 (PTV 1) was generated with a 3D margin of 5 mm around the GTV. PTV 1 was not allowed to overlap with the rectum. PTV 2 was generated by defining a 3D margin of 10 mm around the CTV but only 7 mm in posterior direction.

The bladder (as a solid organ) and both femoral heads were defined as OARs. The rectum was contoured in four different ways: ***1) ***rectal wall based on manual delineation of the inner and outer contour of the rectal wall ***2) ***rectal wall based on manual delineation of the outer contour of the rectal wall and automatic calculation the inner contour using a 3 mm margin [11]***3) ***entire rectal volume including the rectal wall and the rectal lumen ***4) ***rectal surface as the outer contour of the rectal wall. For all four approaches the rectum was confined to 1 cm above to 1 cm below PTV 2 in superior-inferior direction. Therefore, the delineated OAR rectum was different from the anatomical anal canal and rectum as the most superior and inferior parts were not included into the OAR. Anal canal and rectum were not delineated as different OARs to make the analysis and presentation of results more straight-forward [12].

Treatment was planned for a Siemens PRIMUS™ linear accelerator with 6 MV and 18 MV photon energy and a multi-leaf collimator with 1 cm leaf width. The isocenter was placed in the geometrical center of PTV 2. Two 3D-CRT treatment plans were generated for each patient with a prescription dose of 70Gy to PTV 2 according to ICRU 50. Three-field plans with gantry angles of 0° (6 MV), 100° (18 MV) and 260° (18 MV) and four-field plans with gantry angles of 0° (6 MV), 90° (18 MV), 180° (18 MV) and 270° (18 MV) were generated.

A third treatment plan with step-and-shoot IMRT was generated for each patient using optimization objectives listed in Table 1. A simultaneous-integrated boost (SIB) [10] concept with a prescription dose of 66Gy to PTV 2 and a prescription dose of 73Gy to PTV 1 in 33 fractions was applied. Seven beams with 6 MV photon energy were used; the isocentre was placed in the centre of the PTV2. Five intensity levels were allowed for the optimization with a minimum segment size of 2 cm^2 ^and a maximum of 10 segments per beam.

**Table 1:** IMRT optimization objectives for OTP planning system

**Organs-at-risk**				
	Full Volume Dose (Gy)	Max. Dose (Gy)	Over Dose Volume (%)	Limit Dose (Gy)

Bladder	23	50	23	75
Right femoral head	27	41	9	50
Left femoral head	27	41	9	50
Rectum	23	50	21	73

**Target volumes**				

	Min. Dose (Gy)	Prescription Dose (Gy)	Under Dose (%)	Limit Dose (Gy)

PTV 1	68	73	5	81
PTV 2	61	66	5	81

The nomenclature of the OTP TPS was used for description of dose volume objectives: **For organs-at-risk**: the minimum dose should be lower than "full volume dose"; "maximum dose" and "over dose volume" defines one DVH objective; "limit dose" is the maximum dose;

**For targets**: "Under dose (%)" is the volume (%) that is allowed receiving less than the prescription dose; "limit dose" is the maximum dose;

After plan generation the dose distribution was calculated for targets and OARs of each treatment plan. For the rectum the dose distribution to the manually delineated rectal wall (DWH), to the semi-automatic delineated rectal wall with 3 mm wall thickness (DWH_3_) and to the solid rectum including the lumen (DVH) were calculated. The dose distribution to the outer surface of the rectal wall (DSH) was calculated using the CERR software developed at Washington University in St. Louis [13]. Dx (Gy) denotes the minimal dose (Gy) delivered to x volume percent (x area percent for the DSH) of the evaluated volume-of-interest (VOI).

Dose parameters were compared using student's t-test for matched pairs. The Spearman's rank correlation was utilized to test the correlation between pairs of values. For statistical analysis Statistica 6.0 (Statsoft, Tulsa, USA) was utilized. Differences were considered significant for p < 0.05.

## Results

The three-field treatment plans compared with the four-field plans resulted in significantly decreased doses to the rectum in the low dose region D70 and D90. The relationship between rectal DWH_3_, DWH, DVH and DSH was not different between the three-field and the four-field plans. Therefore, only results of the 3-field plans are reported in the following and referred to as 3D-CRT in comparison to results from the IMRT treatment plans.

**Figure 1:**
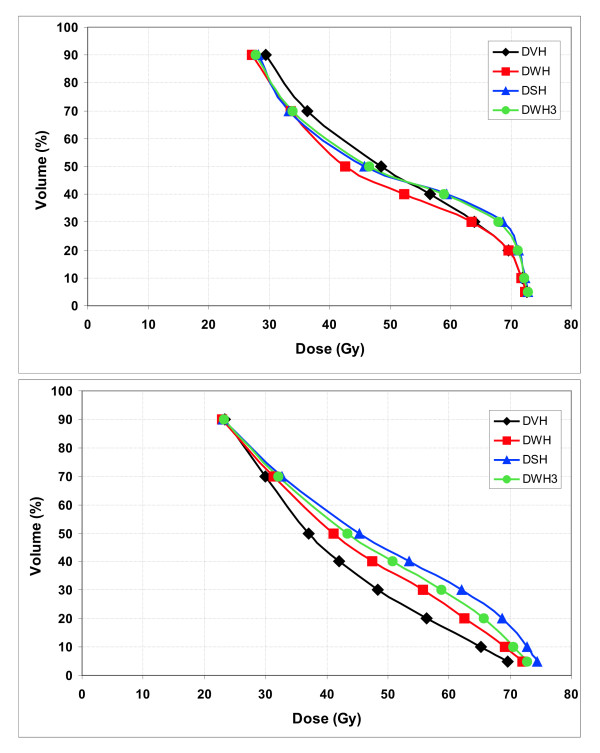
Dose-volume histogram of the rectum (averaged over all n = 10 patients) based on DWH_3_, DWH, DVH and DSH in Fig 1a) 3D-CRT and in Fig 1b) IMRT treatment planning.

The relationship between DWH_3_, DWH, DVH and DSH in 3D-CRT treatment planning is shown in Figure 1a. In the high-dose region D5 to D20 an almost identical dose distribution to the rectum was shown by all four approaches. In the mid-dose region D30 to D50 the doses displayed in the DWH were significantly lower compared to doses in the DSH and the DWH_3_: mean difference of 8.7% ± 4.2% (mean ± SD). In the low-dose region of D70 and D90 the DVH showed significantly higher dose of 6.8% ± 2.2%.

Correlation between corresponding dose parameters was investigated by the nonparametric Spearman's rank test. A highly significant linear correlation between pairs of DWH_3_, DWH, DVH and DSH parameters was shown. Best correlation was seen between DSH and DWH_3 _(R^2 ^= 0.996), worst correlation between DVH and DWH (R^2 ^= 0.939). The slope of linear fit lines ranged between 0.997 (DSH and DWH_3_) and 1.03 (DVH vs. DWH_3_).

Comparing 3D-CRT with IMRT treatment plans, more pronounced differences between dose parameters were seen for the latter (Fig 1b). In IMRT the differences were statistically significant between DVH and all other dose parameters, between DWH and DSH but not between DWH and DWH_3 _and between DSH and DWH_3_. In the high-, mid- and low-dose region the DSH showed significantly higher doses to the rectum compared to the DVH. Doses in the DSH were increased by 23.6% ± 8.7% compared to the DVH in the mid-dose region; differences were smaller in the high-dose region (9.2% ± 6.6%) and in the low-dose region (6.2% ± 3.9%). The DWH showed decreased doses compared with the DWH_3 _in all dose regions.

**Figure 2:**
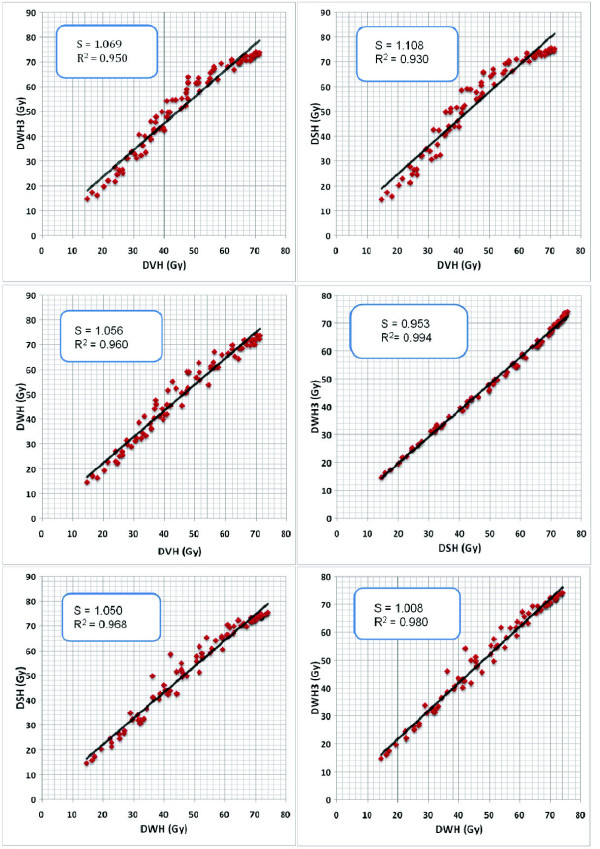
Correlation between dose parameters in IMRT treatment planning. S (slope of linear fit line).

In Fig. 2 the corresponding results of DWH_3_, DWH, DVH and DSH were plotted and linear fit lines were calculated. In general correlation between dose parameters was worse in IMRT plans compared to 3D-CRT plans. Best correlation was seen between DSH and DWH_3 _(R^2 ^= 0.994) and worst correlation between DSH and DVH (R^2 ^= 0.930); the slope of linear fit lines ranged between 1.01 (DWH vs. DWH_3_) and 1.11 (DVH vs. DSH).

The influence of the rectal volume, the degree of rectal filling, on the relationship between the dose parameters was investigated. Patients were equally divided into two sub-groups according to the rectal volume.

**Figure 3:**
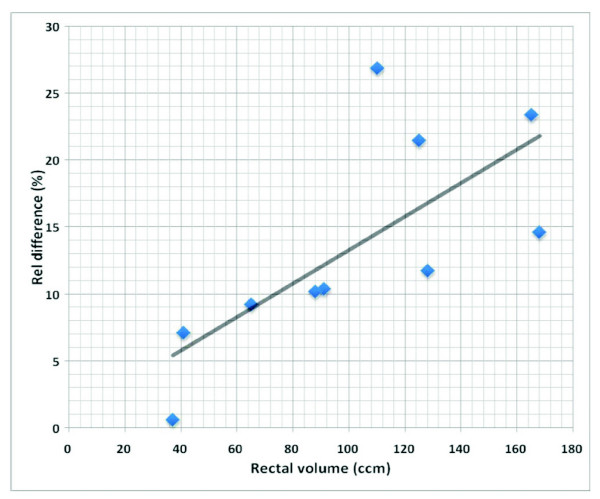
Correlation of rectal volume and relative difference between rectal DVH and DWH in IMRT treatment planning of prostate cancer.

In 3D-CRT treatment planning, no significant difference was seen between DWH_3_, DVH and DSH for patients with small rectal volumes (n = 5). For patients with a distended rectum (n = 5) DSH and DWH showed identical results but DVH showed significantly lower dose to the rectum in the mid-dose region by 6.3% ± 7.2%. In the IMRT treatment plans the influence of the rectal volume on the relationship between dose parameters was different. The order of the dose distribution to the rectum was not different between the sub-groups: DSH > DWH_3 _> DWH > DVH. However, differences between dose parameters were larger in the sub-group with a distended rectum. In the mid-dose region the difference between DVH and DWH was 7.5% ± 3.9% and 19.1% ± 4.9% in the subgroup with small rectal volumes and with a distended rectum, respectively. A statistical significant correlation (r = 0.81) between the rectal volume and the difference between DVH and DWH was observed (Fig 3).

Furthermore, the influence of the anatomy of the seminal vesicles on the relationship between the dose parameters was tested. Two sub-groups were generated with five patients each. The criterion was how far the seminal vesicles were wrapped around the rectum.

In the 3D-CRT plans a significant difference between DSH/DWH_3 _vs. DVH was seen for patients with the seminal vesicles confined to the anterior rectal wall. With the seminal vesicles wrapped around the rectum no difference between DSH, DWH_3 _and DVH was found. Contrary, in the IMRT treatment plans the anatomy of the seminal vesicles influenced the relationship between the dose parameters only marginally.

**Figure 4:**
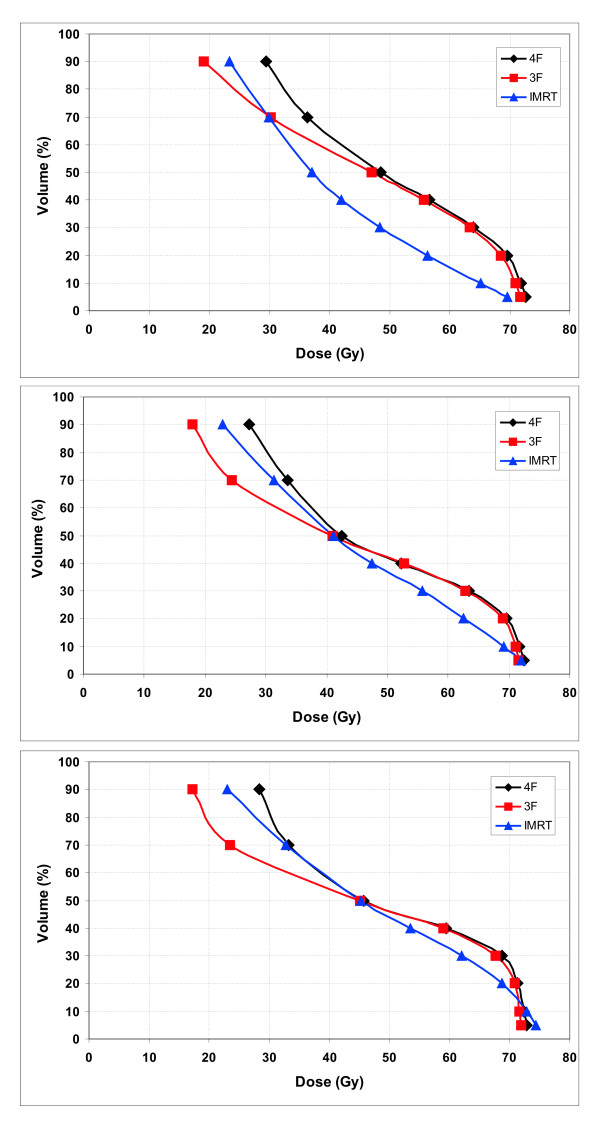
Comparison of 3-field (3F), 4-field (4F) and IMRT treatment plans with the rectal dose based on the DVH (Fig 4a), the DWH_3 _(Fig 4b) and the DSH (Fig 4c).

Dose distribution to the rectum was compared between IMRT and 3D-CRT treatment. Depending on the way of contouring the rectum the benefit of IMRT in sparing the rectum was different (Fig. 4). Comparing IMRT and 3D-CRT the IMRT technique resulted in 23% ± 15% decreased doses to the rectal DVH in the mid dose region. Based on DWH_3 _the benefit of the IMRT technique was 11% ± 11% and based on DSH the benefit was reduced to 7% ± 10%.

## Discussion

Reducing rectal toxicity represents a major challenge in radiotherapy treatment planning for prostate cancer. Treatment with escalated doses was shown to result in improved rates of local control [14,15] but simultaneously higher doses to the rectum were found to be correlated with increased rates of late rectal toxicity. Reliable tools in treatment planning for estimating the risk of toxicity are therefore essential. The dose-volume histogram is a common tool to express the dose that is delivered to targets and OARs. Though dose-volume histograms do not provide spatial information, i.e. the location of the high- and low-dose regions ("hot" and "cold" spots) inside the volume of interest, multiple studies have shown correlation between dose-volume-histogram parameters and rectal toxicity. In table 2 a literature review about these studies is given.

**Table 2:** Literature review of dose-volume relationship for late rectal bleeding in radiotherapy of prostate cancer

**Author**	**Patients**	**Follow up**	**Persription Doses**	**Treatment technique**	**Classification of toxicity**	**Endpoint**	**Events**	**Dosimetric parameter**	**Rectum delineation**	**Results**
Hartford 1996 [29]	41	Minimum 4 years	50.4Gy 25.2CGE	4 field Perineal proton boost	RTOG	≥ Grad I rectal bleeding	14	DWH ant. RW	From superior limit of anus to 2 cm superior to prostate	Cut-off: Continuously between 60Gy to 70% and 75Gy to 30%

Boersma 1998 [30]	130	Median 24 months	70 – 76Gy	3 field 3D-CRT	SOMA/LENT and RTOG/EORTC	≥ Grad III rectal bleeding	2	DWH	15 mm caudal to the apex of the prostate to boarder to sigmoid	Cut-off: ≥ 65Gy to >40% ≥ 70Gy to >30% ≥ 75Gy to >5% (no correlation for grade I/II rectal bleeding)

Storey 2000 [31]	189	Minimum 2 years	70Gy 78Gy	4 field box 4 field box, 6 field 3D-CRT boost	Modified RTOG	≥ Grad II late rectal toxicity	28	DVH	Rectum included within 11 cm of initial APPA field	For patients treated to 78Gy: Cut-off: ≥ 70Gy to >25%

Jackson 2001 [32]	451	Minimum 30 months	70.2Gy 75.6Gy	6 field arrangement 3D-CRT	RTOG	≥ Grad III late rectal bleeding	49	DWH	below sigmoid flexure to above anal verge	Correlation with: # area under the average percent volume DWH # Exposure to ~62% and to ~102% of prescription dose

Fenwick 2001 [33]	79	Minimum 2 years	60 – 64Gy	3 field • 3D-CRT • Conventional	RTOG	Grade I – III rectal bleeding	?	DSH	up to level of rectosigmoid junction	Correlation with: % of RS exposed to > 57Gy

Wachter 2001 [34]	109	Median 30 months	66Gy	4 field 3D-CRT	EORTC/RTOG	Grade II rectal bleeding	15	DVH	From lower to upper boarder of 4 field	Cut-off: ≥ 60Gy to >57%

Kupelian 2002 [35]	128	Median 24 months	78Gy 70Gy	4 field (42Gy) 6 field boost (36Gy): 3D-CRT IMRT (SD 2.5Gy)	RTOG	Grade I – III rectal bleeding	9	DVH	From 1 cm above to 1 cm below the target	Cut-off: Absolute rectal volume: ≥ 78Gy to >15 cm3

Huang 2002 [36]	163	Median 62 months	74 – 78Gy	4 field conventional (46Gy) 6 field boost 3D-CRT	Modified RTOG	≥ Grad II late rectal toxicity	38	DVH	11 cm in length starting at 2 cm below the inferiormost aspect of the ischial tuberosities	Cut-off: V60 below 40% V70 below 25% V75.6 below 15% V78 below 5%

Fiorino 2003 [37]	245	Median 2 years	70 – 78Gy	3 to 4 field 3D-CRT	Modified RTOG	Grade II – III rectal bleeding	23	DVH	Above anal verge to sigmoid	Cut-off: V50 below 60–65% V60 below 50–55% V70 below 25–30%

Greco 2003 [38]	135	Median 28 months	76Gy	6 field 3D-CRT	RTOG	≥ Grad II late rectal toxicity	24	DVH	from just below the sigmoid flexure to just above the anal verge	Cut-off: V40 below 60% V50 below 50% V60 below 25% V72 below 15% V76 below 5%

Akimoto 2004 [39]	52	Median 31 months	69Gy SD 3Gy	unblocked 4 field technique to the prostate	RTOG	≥ Grad II late rectal toxicity	13	DVH	above anal verge to point at which it turns into the sigmoid colon	Cut-off (equivalent 83Gy prescription dose): V30 (V24.9) to ≥ 60% V50 (V41.5) to ≥ 40% V80 (V66.4) to ≥ 40% V90 (V74.7) to ≥ 15%

Koper 2004 [40]	266	Minimum 2 years	66Gy	Conventional (n = 125) 3 field 3D-CRT (n = 123)	RTOG	≥ Grad I late rectal toxicity	57% 47%	DVH (separately for proximal, middle and distal part of rectum)	length of intestinal structures was limited to cranial and caudal field borders	Correlation with: Distal rectal volume exposed to ≥ 90% tumor dose

Lee 2005 [41]	212	Median 86 months 35 months	66 70 – 74Gy	Conventional 3D-CRT	Modified RTOG/Lent and RTOG	≥ Grad II late rectal toxicity	34	DVH	?	Cut-offs: ≥ 60Gy to >51.5% ≥ 70Gy to >41.5%

Vargas 2005 [11]	331	Median 19 months	70.2Gy to 79.2Gy	Adaptive 3D-CRT	CTC 2.0	≥ Grad II late rectal toxicity	43	DVH, DWH	from the anal verge or ischial tuberosities (whichever was higher) to the sacroiliac joints or rectosigmoid junction (whichever was lower)	Association with: DWH: V50, V60, V66.6, V70, V72 DVH V60–V72

Peeters 2006 [42]	614	Median: 44 months	68Gy vs 78Gy	3D-CRT	Adapted RTOG/EORTC	≥ Grad II rectal bleeding	31	DWH	anorectal, rectal, and anal wall dose volume histogram	Correlation with: anorectal V55–V65

However, the transfer of the results from table 2 into clinical practice is complicated by the different way of contouring the rectum, different toxicity endpoints and different classifications of rectal toxicity in the literature.

Within this retrospective planning study it was demonstrated that the method of contouring the rectum significantly influenced the "dose to the rectum" represented in the dose-volume histogram. In general, delineation of the rectal volume as a solid organ underestimated the exposure of the rectum compared to delineation of the rectal surface or the rectal wall. The differences were larger in IMRT treatment planning compared to 3D-CRT. For one single patient the dose to the rectum in the mid-dose region was 35% higher in the DSH compared to the DVH. The rectum was delineated from 1 cm superior to 1 cm inferior the PTV. The delineated OAR rectum constituted a fairly constant fraction of the anatomical anus/rectum averaged over all patients (73% ± 4%). Portions of the rectum outside the beam, receiving very low doses, were therefore excluded from analysis. Differences between dose parameters would have been smaller if the complete anatomical anus and rectum would have been contoured.

It was also demonstrated that there was no constant relationship between dose parameters DWH_3_, DWH, DVH and DSH for all patients. Both the rectal volume, the degree of the rectal filling, and the anatomy of the seminal vesicles were shown to be relevant. The pattern how these anatomic characteristics influenced the relationship between DWH_3_, DWH, DVH and DSH was different in IMRT and 3D-CRT treatment planning. Because of significant differences between dose parameters and because dose volume histograms do not provide spatial information the importance of reviewing the dose distribution in every single CT slice and not only relying on dose parameters has to be stressed.

Others studies compared rectal DVH, DWH and DSH in treatment planning of the prostate [16-20]. Using a cylindrical model for the rectum Fiorino et al. described substantial differences between DVH and DWH for a "full" rectum but only small differences for an "empty" rectum. For patients with a distended rectum the DSH was close to the DWH. Boehmer et al. [20] showed that the length of delineating the rectum in superior-inferior direction significantly influenced the dose to the rectum and therefore should be standardized. However, all these studies are based on 3D-CRT. In this work it has been clearly demonstrated that a one-to-one transfer of the results from 3D-CRT to IMRT treatment planning is not possible.

Another interesting result of this study was the finding that the dose to the manually delineated rectal wall (DWH) was different from the dose to the semi-automatically generated rectal wall with 3 mm wall thickness (DWH_3_). The choice of the 3-mm wall thickness is supported by the study of Rasmussen, in which the rectal wall thickness measured by ultrasound was found to have a median of 2.6 mm [21]. Tucker et al. reported only small differences of the DWH for rectal wall thicknesses ranging between 2 mm and 5 mm [19]. As the patients in this study were treated in a supine position the intra-rectal feces moved to the posterior rectal wall due to gravity. With CT density values of the rectal wall often very similar to the density of the filling a precise delineation of the inner contour of the rectal wall was difficult for some patients resulting in asymmetric rectal wall thicknesses between anterior (within high-dose region) and posterior (within mid- to low-dose region) rectal wall. It is likely that this explains the differences between DWH_3 _and DWH and because of this difficulty and uncertainty we do not advocate delineating the inner contour of the rectum manually. Though automatic generation of the DWH_3 _reduced uncertainties compared to DWH, the thickness of the rectal wall is dependent on the rectal distension and consequently not constant. Meijer et al. described a more sophisticated method of automatic DWH generation [18]: based on the delineated outer rectal contour the inner contour was generated automatically taking the rectal distension into account.

Delineation of the outer contour of the rectum was found to be associated with small intra- and inter-observer variability [22,23]. Consequently, in analysis of DVH and DSH uncertainties are expected to be lower compared to DWH analysis. Furthermore, generation of the DSH and the DVH are known to be sensitive to parameters such as voxel dimensions and dose calculation grid size [16]. These facts could partially be responsible for differences between dose parameters.

Recently, de Crevoisier et al. showed an increased risk of local failure and simultaneously a lower incidence of late rectal bleeding for patients with a distended rectum on the planning CT study [9]. Treatment planning based on a planning CT with distended rectum introduced a systematic error with the prostate and the anterior rectal wall moving posterior out of the high-dose-region during the treatment. Repetition of the planning CT study in case of a distended rectum was suggested to avoid this error. Additionally, good agreement between DVH and DWH was shown in case of an empty rectum making transfer of constraints form the literature to treatment planning more reliable.

The fact that one single planning CT study is only a snapshot of the patients' anatomy has to be considered for the interpretation of dose-volume histograms. Image-guided treatment techniques are thought to correct differences between treatment planning and the current anatomy at the time of treatment [24-27]. Recently, technologies introduced 3D volume imaging into the treatment room with sufficient soft-tissue contrast for visualization of the prostate and OARs [28]. Such image-guided treatment protocol are expected to allow a substantial reduction of safety margins and consequence in a further escalation of the treatment dose [25].

Comparison of 3D-CRT and IMRT in terms of sparing the rectum was not aim of this study. A simultaneous integrated boost concept was applied for the IMRT plans whereas a homogenous dose distribution without field size reduction was planned for the 3D-CRT plans. It was interesting to note that the "benefit" of IMRT in comparison to 3D-CRT was strongly dependent on the way of contouring the rectum. Doses to the rectum were reduced in the IMRT plan by 23%, 11% and 7% with the calculation based on the rectal DVH, DWH_3 _and the DSH.

## Conclusion

This study demonstrated that the method of delineating the rectum significantly influenced the dose representation in external beam radiotherapy of localized prostate cancer. Differences between the dose parameters, based on delineation of the rectal wall, rectal volume and rectal surface, were larger in IMRT treatment planning compared with 3D-CRT. It was shown that the patient's anatomy, both the rectal filling and the anatomy of the seminal vesicles, influenced the relationship between the four evaluated parameters. For integration of dose-volume parameters from the literature into treatment planning these results have to be considered: a one-to-one transfer of the results from 3D-CRT to IMRT treatment planning may be associated with substantial errors.

## Competing interests

The author(s) declare that they have no competing interests.

## Authors' contributions

All authors read and approved the final manuscript.

MG designed the analysis, generated the treatment plans, performed the analysis and drafted the manuscript.

JM was involved in the statistical analysis and revised the manuscript.

KB participated in the study design and revised the manuscript.

DV participated in the study design and revised the manuscript.

MF participated in the study design and revised the manuscript.
